# Comparative Analysis of Morphology, Photosynthetic Physiology, and Transcriptome Between Diploid and Tetraploid Barley Derived From Microspore Culture

**DOI:** 10.3389/fpls.2021.626916

**Published:** 2021-02-26

**Authors:** Yunyun Chen, Hongwei Xu, Ting He, Runhong Gao, Guimei Guo, Ruiju Lu, Zhiwei Chen, Chenghong Liu

**Affiliations:** ^1^College of Fisheries and Life Science, Shanghai Ocean University, Shanghai, China; ^2^Biotechnology Research Institute, Shanghai Academy of Agricultural Sciences, Shanghai, China; ^3^Biotechnology Research Institute, Shanghai Key Laboratory of Agricultural Genetics and Breeding, Shanghai, China

**Keywords:** barley (*Hordeum vulgare* L.), tetraploid, photosynthesis, RNA-seq, quantitative PCR

## Abstract

Polyploids play an important role in the breeding of plant for superior characteristics, and many reports have focused on the effects upon photosynthesis from polyploidization in some plant species recently, yet surprisingly little of this is known for barley. In this study, homozygous diploid and tetraploid plants, derived from microspore culturing of the barley cultivar “H30,” were used to assess differences between them in their cellular, photosynthetic, and transcriptomic characteristics. Our results showed that tetraploid barley has the distinct characteristics of polyploids, namely thicker and heavier leaves, enlarged stomata size or stomatal guard cell size, and more photosynthetic pigments and improved photosynthesis (especially under high light intensity). This enhanced photosynthesis of tetraploid barley was confirmed by several photosynthetic parameters, including net photosynthetic rate (P_n_), stomatal conductance (G_s_), intercellular CO_2_ concentration (C_i_), transpiration rate (T_r_), maximum net photosynthetic rate (P_max_), light saturation point (LSP), maximum RuBP saturated rate carboxylation (V_cmax_), and maximum rate of electron transport (J_max_). Transcriptomic analyses revealed that just ~2.3% of all detected genes exhibited differential expression patterns [i.e., differentially expressed genes (DEGs)], and that most of these – 580 of 793 DEGs in total – were upregulated in the tetraploid barley. The follow-up KEGG analysis indicated that the most enriched pathway was related to photosynthesis-antenna proteins, while the downregulation of DEGs was related mainly to the light-harvesting cholorophyII *a*/*b*-binding protein (Lhcb1) component, both validated by quantitative PCR (qPCR). Taken together, our integrated analysis of morphology, photosynthetic physiology, and transcriptome provides evidences for understanding of how polyploidization enhances the photosynthetic capacity in tetraploids of barley.

## Introduction

Haploids are the fundamental ploidy-level type, from which diploids or tetraploids are easily produced *via* chromosome doubling. Microspores as gametic cells that have undergone androgenesis are able to produce haploid plants through *in vitro* culturing. This microspore culture provides a useful way for plant breeders to generate genetically stable, homozygous diploids, and colchicine is almost always used to induce the chromosome doubling. Furthermore, plant chromosomes’ doubling can spontaneously occur during anther or microspore culturing (Yuan et al., 2015). For instance, spontaneous chromosome doubling was reportedly higher than 50% in cabbage, broccoli, and barley (Castillo et al., 2009; Yuan et al., 2015). Hence, polyploidization events are very frequent in anther or microspore culture when using colchicine for chromosome doubling, even for wheat plants (Soriano et al., 2007).

Polyploid plants are generally superior to diploid plants in terms of morphology, metabolite contents, and tolerance to biotic or abiotic stresses (Sattler et al., 2016), and the current consensus is that photosynthetic capacity is the dominant characteristic of polyploid plants (Arias and Bhatia, 2015). A plant’s photosynthetic capacity is usually associated with its leaf thickness, stomatal size, and composition of photosynthetic pigments (Liu et al., 2018). Previous studies have shown that polyploids can have thicker leaves than diploids (Sun et al., 2015), polyploidization increases stomatal size (Laere et al., 2011; Zhang et al., 2017), and tetraploid plants of *Chrysanthemum nankingense* harbor significantly higher chlorophyll contents than counterpart diploids (Dong et al., 2017). However, in comparison with diploids, the changed photosynthetic rate in polyploids differs according to the species studied. For example, it is higher in *Allium oleraceum* (Eliška et al., 2015), *Phlox drummondii* (Vyas et al., 2007), *Malus* × *domestica* (Xue et al., 2017), and *Lilium* (Cao et al., 2018), but lower in *Triticum* (Hejnák et al., 2016) and *Fragaria* (Gao et al., 2017), yet moderate in *Hordeum vulgare* (Sicher et al., 1984).

Barley (*H. vulgare* L.), a worldwide staple crop, has been used for food, feed, brewing, and health products (Newman and Newman, 2006; Gao et al., 2018). Due to its high frequency of embryogenesis, barley has also been thought of as an excellent model plant for breading double haploids (Lu et al., 2008). In our prior work, we obtained both diploid and tetraploid barley plants from microspore cultures, finding that the latter possess thicker leaves than the former. Yet, an early study found no significant differences in the net photosynthetic rate between diploid and tetraploid barley plants under controlled environment conditions (Sicher et al., 1984). Since then, however, we could not find other published work that updates or verifies that finding. Moreover, little is still known of molecular mechanisms responsible for differences between the diploid and tetraploid barley plants.

In this study, we characterized the differences in morphological and photosynthetic parameters between the tetraploids and diploids of barley. Further, a transcriptome analysis was performed to investigate the distinct patterns of gene expression between the plant types. Our aim was to evaluate the effects of polyploidization upon photosynthetic capacity by comparing the phenotypic, photosynthetic, and molecular characteristics between tetraploids and diploids of barley, and to uncover the potential superiority of tetraploids in polyploid barley breeding.

## Materials and Methods

### Plant Materials and Growth Conditions

The double haploid and tetraploid barley were obtained from a microspore culture of the barley cultivar “H30,” which is the one primarily used in China’s Shanghai region for malt barley production (Chen et al., 2012; Gao et al., 2018). The plant growth conditions of these barley plants are detailed by Xu et al. (2016). Briefly, the seeds were sterilized in 1% (v/v) hypochlorite solution for 30 min, and then germinated in a phytotron at 22 ± 2°C for 1 week. Plants at approximately the two-leaf stage were transferred into plastic tanks containing Hoagland solution, in the phytotron of Shanghai Academy of Agricultural Sciences. The solution of each tank was replaced every 2 days and its pH maintained at 6.2 ± 0.2. Shoots of diploid and tetraploid barley were harvested at 11:30 am, and one part was used for investigation of shoot dry weight according to Xu et al. (2016) and the other part was used for their RNA isolation, for which samples were rapidly frozen in liquid nitrogen and stored at −80°C.

### Identification of the Diploid and Tetraploid Barley Plants

To distinguish diploid and tetraploid barley, the counting of chromosome number and measuring of stomatal guard cells’ length were relied upon. For chromosome counting, three new root tips with a length of 1–2 cm were cut from roots of each barley seedling (at the two-leaf stage), and then incubated in a precooled 90% glacial acetic acid for ca. 10 min (Hu et al., 2015). Next, the root tips were dried with absorbent paper, and then immersed in 70% ethanol and stored at −20°C for later use. Finally, root tips were dissociated in 45% acetic acid, for 2 h, and then observed under a microscope (Olympus BX51, Tokyo, Japan). To measure the length of stomatal guard cells, the middle part of the top second leaf, having a length of 3–4 cm, was sampled and soaked in a Carnoy’s fixative solution [its ratio of anhydrous ethanol to glacial acetic acid was 3:1 (v/v)] until all leaf sections were completely discolored (He et al., 2014). Finally, they were rinsed with distilled water, and the lengths of stomatal guard cells of closed stomas were measured under a 400× field Olympus microscope (Olympus DP71, Tokyo, Japan). Five stomas per leaf section were randomly selected and measured in this way, from 11 different leaves of diploid or tetraploid barley plants, respectively, for a total of 55 replicates for each. The stomatal density was estimated by counting the number of stomata in each field under Evos FL Auto 2 (Invitrogen) with parameters of Objective: 20×, Light source: Trans, Mode: simple, and Camera: color. Five observation fields per leaf were evenly distributed in each leaf, and there were three different leaves of diploid or tetraploidbarley plants, respectively, for a total of 15 replicates for each.

### Photosynthetic Pigment Extraction and Quantification

The extraction of photosynthetic pigments was mainly according to Arnon (1949), Porra (2002), and Cao et al. (2018). Briefly, the fresh (top) second leaves of diploid and tetraploid barley plants were cut into pieces, and then submerged in 80% (v/v) acetone for 3–5 min, in the dark, at room temperature. Then, their chlorophyll *a* (Chl *a*), chlorophyll *b* (Chl *b*), and carotenoid (Car) contents were measured with a spectrophotometer (PuXi Tong Yong T6 new century, Beijing, China) at 663, 645, and 470 nm, respectively. Three biological replicates were used for each sample.

### Measurement of Photosynthetic Parameters

Five photosynthetic parameters, namely net photosynthetic rate (P_n_) estimated by CO_2_ uptake (it is also represented by “A” in the equipment), stomatal conductance (G_s_; it is also represented by “gsw” in the equipment), intercellular CO_2_ concentration (C_i_) and transpiration rate (T_r_; it is also represented by “E” in the equipment), were each measured by a LI-6800 portable photosynthesis system (LI-COR, United States; Farquhar et al., 1980). To assess diurnal variation in photosynthesis, measurements were taken every 2 h from 7:30 am to 5:30 pm under natural light conditions on a sunny day. The light- and CO_2_-response curves were constructed by using photosynthesis v1.0 software (Long and Bernacchi, 2003). The maximum net photosynthetic rate (P_max_) estimated by CO_2_ uptake (it is also represented by “Amax” in the software), light saturation point (LSP) and light compensation point (LCP), and apparent quantum efficiency (AQE) were calculated from the light-responsive curve. The maximum RuBP saturated rate of carboxylation (V_c,max_) and maximum rate of electron transport (J_max_) were calculated from the CO_2_-response curve. The fluorescence was measured automatically, and the maximal photochemical efficiency of PSII in light (F_v_’/F_m_’) was recorded directly using the LI-6800. The top second leaf was used for all measurements above, with 10 biological replicates for each sample.

### RNA Isolation, cDNA Library Construction, and Sequencing

Total RNA was isolated by using the Trizol Reagent (Invitrogen, Carlsbad, CA, United States) and following its manufacturer’s instructions. Each sample’s RNA concentration and quality were measured by a Nano Drop spectrophotometer (Thermo Scientific). The RNA integrity was evaluated with a 2,100 Bioanalyzer (Agilent Technologies, Palo Alto, CA, United States). To construct each cDNA library, 3 μg of RNA was used and its sequencing was performed on a Hi-Seq platform (Illumina) by Shanghai OE Biotech CO., Ltd., for which 150 bp paired-end reads were generated. Three biological replicates were used for each sample.

### Identification of DEGs and Their Functional Analysis

Clean data were obtained by removing those reads containing adapter sequences and any low-quality reads from the raw data, using the Trimmomatic tool (Bolger et al., 2014). Both Q30 and GC information variables were calculated to evaluate the cleaned dataset, for which clean nucleotide sequence data ranged from 6.73 to 6.87 Gb (all >6 Gb), and the Q30 percentages were all >90% (Supplementary Table S1). Spearman correlations and principal component analysis (PCA) further showed that the three biological replicates per sample met the requirements for a robust analysis (all over 0.96; Supplementary Figure S1). The clean reads were aligned to the reference genome using the hisat2 tool (Kim et al., 2015), and barley’s genome data were directly downloaded from the Ensembl Plants website.^1^ Putative transcript annotations were performed by searching the listed annotations of high confidence (HC) genes.^2^ The mapped reads and gene expression level were assembled using the htseq-count script (Anders et al., 2015) and Cufflinks (Roberts et al., 2011), respectively. The fragments per kilo bases per million reads (FPKM) was used to evaluate the level of gene expression. The differentially expressed genes (DEGs) were identified by DESeq (v1.26.0, European Molecular Biology Laboratory, Heidelberg, Germany), as described by Li et al. (2019), designated as those genes with a threshold fold change (FC) ≥ 2 and an *p* < 0.05. Hierarchical analysis was applied to these DEGs in Cluster 3.0 software after their normalization. Gene Ontology (GO) and Kyoto Encyclopedia of Genes and Genomes (KEGG) enrichment analysis of the DEGs (Simon Anders) were used to predict their respective functions by searching the GO^3^ and KEGG^4^ databases.

### Validation by qRT-PCR

To validate the RNA-Seq data and confirm their expression profiles, all DEGs involved in the pathway of photosynthesis-antenna proteins were selected for a qRT-PCR analysis, since this pathway was the most enriched one. The sequences of these genes were directly downloaded from the Ensembl Plants website. The primers were designed by using Primer-BLAST in NCBI, and thee can be found in Supplementary Table S2. The first-strand cDNA was synthesized with the PrimeScript™ II 1st Strand cDNA Synthesis Kit (Takara, Japan), according to the manufacturer’s instructions. The quantitative PCR (qPCR) reactions were performed on the 7,500 Fast platform (Applied Biosystems, United States) using the PowerUp™ SYBR™ Green Master Mix (Applied Biosystems). Each 20-μl reaction contained 10 μl of the PowerUp™ SYBR™ Green Master Mix, 2 μl of 5× diluted cDNA, 0.8 μl of each forward and reverse primer (10 μM). Each reaction was pre-denatured at 50°C for 20 s, and then at 95°C for 10 min, followed by 40 cycles of 95°C for 15 s and 60°C for 1 min. The melting curves were conducted after these 40 cycles. A given gene’s expression level was first normalized according to a reference gene, *HvGAPDH* (Quan et al., 2016), and its amplification efficiency evaluated in LinReg software (Ramakers et al., 2003). Three biological replicates were used for each qPCR, for which the data analysis and statistics were carried out as described by Chen et al. (2013).

### Statistical Analysis of Experimental Data

To test for differences in the means of stomatal guard cell length, chlorophyll content, photosynthetic variables, and gene expression levels’ data by qPCR, the Student’s *t*-test was used in MS Excel 2010 or Fisher’s least significant difference (LSD) test was implemented in SPSS 21.0 software.

## Results

### Identification of Diploid and Tetraploid Barley Plants

**Figure 1:**
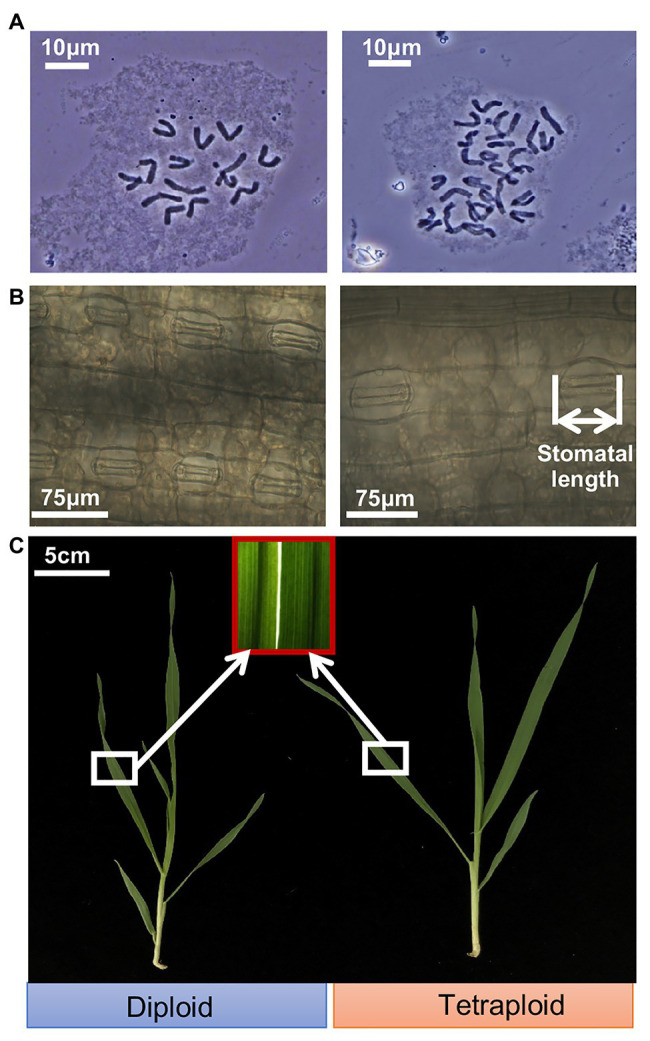
Ploidy analysis and appearance of shoots of the diploid and tetraploid barley seedlings. **(A)** Chromosomes of the diploid (2*n* = 14) and tetraploid (2*n* = 28) barley at mitotic metaphase. **(B)** Closed stomas and straightened stomatal guard cells. **(C)** Shoots of the diploid and tetraploid barley seedlings.

Cytological anatomy analysis confirmed the presence of 14 chromosomes (2*n* = 2*x* = 14) for the diploid barley plants, and corresponding doubled chromosomes (2*n* = 4*x* = 28) for the tetraploid barley plants (Figure 1A). The average length of stomatal guard cells of tetraploid barley (71.0 ± 7.3 μm) was greater than that of diploid barley (49.3 ± 3.3 μm), while the stomatal density was in the opposite situation (the stomatal density of dipoid barley was 35.7 ± 5.8 stomatas per field, while the tetraploid was 20.5 ± 3.2 stomatas per field; Figure 1B; Supplementary Figure S2). It seemed that leaves were thicker in the tetraploid than diploid barley plants by color and texture, and it was consist with the trait of shoot dry weight (Figure 1C; Supplementary Table S3). While the diploid barley grew more rapidly (reaching the 5-leaf stage) than did the tetraploid barley (it reached the four-leaf stage only; Figure 1C).

### Photosynthetic Pigment Contents

This analysis revealed that all the pigments – chlorophyll *a* (Chl *a*), chlorophyll *b* (Chl *b*), total chlorophyll [Chl (*a+b*)] and carotenoid (Car) contents – were significantly higher in the tetraploid barley than diploid barley plants (Table 1). According to these differences, the rank order of increased percentage for the four pigments was Chl *a* > Chl (*a+b*) > Chl *b* > Car.

**Table 1:** Photosynthetic pigment contents in the diploid and tetraploid barley leaves.

Ploidy	Chl *a* (μg ml^−1^)	Chl *b* (μg ml^−1^)	Chl (*a+b*) (μg ml^−1^)	Car (μg ml^−1^)
Diploid	0.39 ± 0.09b	0.13 ± 0.03b	0.52 ± 0.12b	0.28 ± 0.07b
Tetraploid	0.55 ± 0.04a	0.18 ± 0.01a	0.73 ± 0.05a	0.37 ± 0.03a
Difference (%)	41.03	38.46	40.38	32.14

Values represent the mean ± SD (*n* = 3). Different letters indicate significant differences between the diploid and tetraploid barley leaves for each photosynthetic pigment.

### Diurnal Variation of Photosynthesis

**Figure 2:**
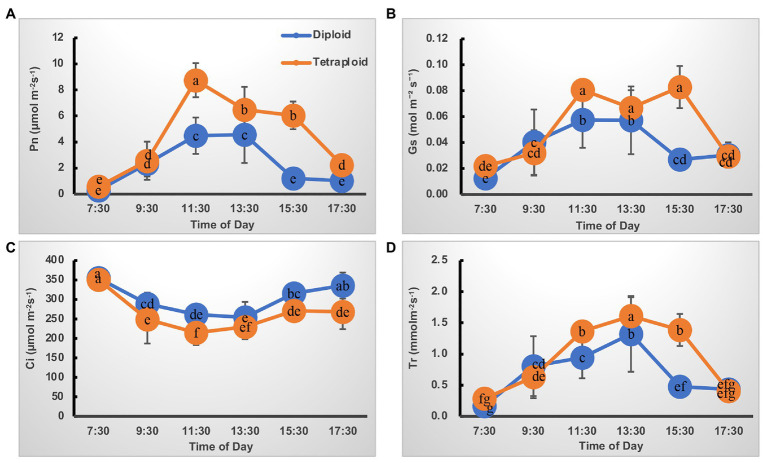
Diurnal variation of photosynthetic characteristics of the diploid and tetraploid barley plants. **(A)** Net photosynthetic rate (Pn). **(B)** Stomatal conductance (Gs). **(C)** Intercellular CO_2_ concentration (Ci). **(D)** Transpiration rate (Tr). Different letters represent significant differences (of Pn or Tr or Gs or Ci) between diploid and tetraploid barley at different time points (*n* = 10).

The trend in diurnal variation of photosynthesis was quite similar in the diploid and tetraploid barley plants (Figure 2). For the net photosynthesis rate (P_n_) curve, its values increased from 7:30 amonward, peaking at 11:30 am, but then gradually declined until 5:30 pm in the tetraploid barley (Figure 2A). Although the P_n_ of diploid barley likewise peaked at 11:30 am, a plateau was apparent at noon as well. In addition, the P_n_ of tetraploid barley exceeded that of diploid barley at each time point after 9:30 am. Concerning the stomatal conductance (G_s_) curve, it closely matched the Pn curve except for a plateau in tetraploid barley lasting from 11:30 pm to 3:30 PM (Figure 2B), and the G_s_ was higher in tetraploid than diploid barley at all sampled times. Similarly, the transpiration rate (T_r_) curve followed the same trend in both diploid and tetraploid barley, but with peaks at 3:30 pm and being higher in the tetraploid barley at the three time points (11:30 am to 3:30 pm; Figure 2D). Unlike those three curves, intercellular CO_2_ concentration (C_i_) first gradually declined, but after reaching its lowest point, it increased gradually (Figure 2C). However, the C_i_ of tetraploid barley increased somewhat later in the daytime than did diploid barley, and the C_i_ values of diploid barley at all the time points except 7:30 am and 1:30 pm were significantly higher than those of tetraploid barley.

### Response of Photosynthesis to Irradiation and CO_2_ Concentration

**Figure 3:**
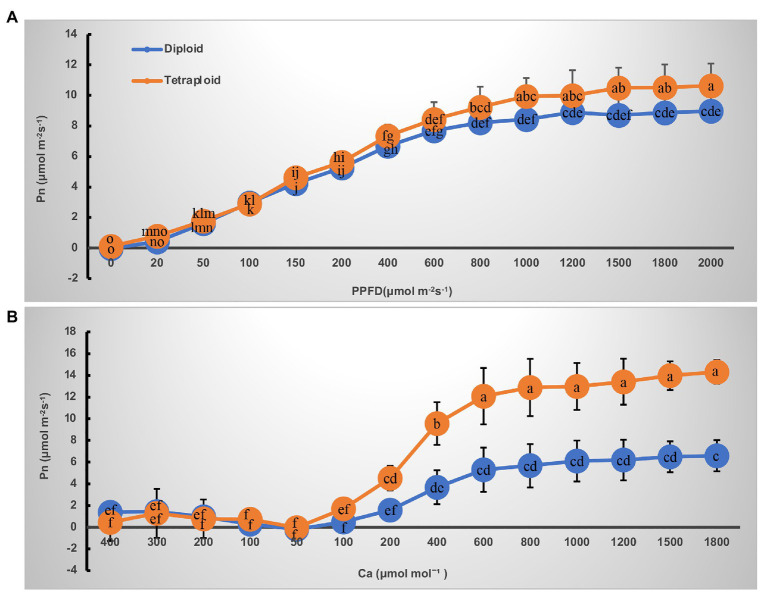
Curves of net photosynthetic rate (Pn) response to irradiance and CO_2_ in leaves of the diploid and tetraploid barley. **(A)** Response to photosynthetic photon flux density (PPFD). **(B)** Response to ambient CO_2_ concentration (Ca). Different letters represent significant differences of Pn between diploid and tetraploid barley under varying supply of PPFD or Ca (*n* = 10).

The light- and CO_2_-response curves of diploid and tetraploid barley are presented in Figure 3. With greater light irradiation and higher CO_2_ concentration, the P_n_ increased rapidly before 1,000 μmol m^−2^ s^−1^ of photosynthetic photon flux density (PPFD) and 600 μmol mol^−1^ of CO_2_, respectively, and then each plateaued. The tetraploid barley showed a significantly higher Pn than did diploid barley when PPFD attained 1,000 μmol m^−2^ s^−1^ or more (Figure 3A), and also a significantly higher P_n_ than the diploid barley when ambient C_a_ was at least 200 μmol mol^−1^ (Figure 3B). Based on the fitted light-and CO_2_-response curves, all four parameters – maximum net photosynthetic rate (P_max_), light saturation point (LSP), maximum rate of RuBP carboxylation (V_c,max_) and maximum rates of electron transport (J_max_) – were significantly higher in the tetraploid than diploid barley leaves (Table 2). The light compensation point (LCP), apparent quantum yield (AQY), and maximal photochemical efficiency of PSII in light (F_v_’/F_m_’) were similar between the diploid and tetraploid barley plants (Table 2).

**Table 2:** Leaf photosynthetic properties in the diploid and tetraploid barley.

Ploidy	P_max_	LSP	LCP	AQE	V_c,max_	J_max_	F_v_’/F_m_’
	(μmol m^−2^ s^−1^)						
Diploid	9.23 ± 1.01b	760 ± 91.20b	8 ± 0.96a	0.033 ± 0.00a	14.34 ± 2.01b	173.93 ± 24.35b	0.26 ± 0.06a
Tetraploid	10.38 ± 0.93a	932 ± 88.54a	9 ± 0.02a	0.029 ± 0.00a	29.42 ± 3.82a	343.19 ± 41.18a	0.23 ± 0.04a

Values represent the mean ± SD (*n* = 10). Different letters indicate significant differences between the diploid and tetraploid barley leaves in each photosynthetic property.

### Differentially Expressed Genes of Tetraploid Barley vs. Diploid Barley

**Figure 4:**
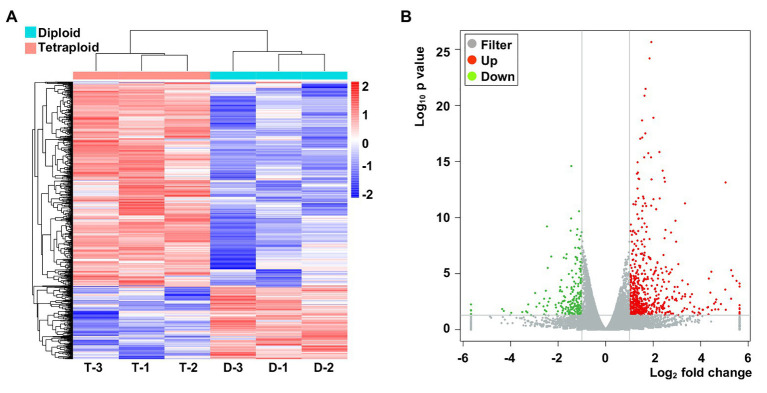
The abundance of specifically expressed mRNA transcripts in the six barley samples and differentially expressed genes (DEGs). **(A)** Cluster of specifically expressed mRNAs, for which D-1,2,3 denote three biological replicates of diploid barley; T-1,2,3 denote three biological replicates of tetraploid barley. **(B)** Volcano of DEGs in the tetraploid barley comparing to the diploid barley.

Considering the higher photosynthesis activity at noontime and the higher P_n_ in the tetraploid barley, samples were taken at ca. 11:30 AM for the RNA-Seq (these data were deposited with NCBI under submission ID: SUB7735025 and bioProject ID: PRJNA642324). The normalized expression of mRNAs was compared between the diploid and tetraploid barley. The clustering results showed that genes grouped into two categories in the diploid and tetraploid barley samples (Figure 4A). In total, 793 genes displayed differential expression levels between diploid and tetraploid barley, of which 580 were upregulated and 213 were downregulated in tetraploid barley vis-à-vis diploid barley (Figure 4B). These DEGs were found located across all barley chromosomes yet they were most abundant on Chromosome 2 (Supplementary Material of DEGs).

### Functional Analysis of DEGs in the Tetraploid Barley

**Figure 5:**
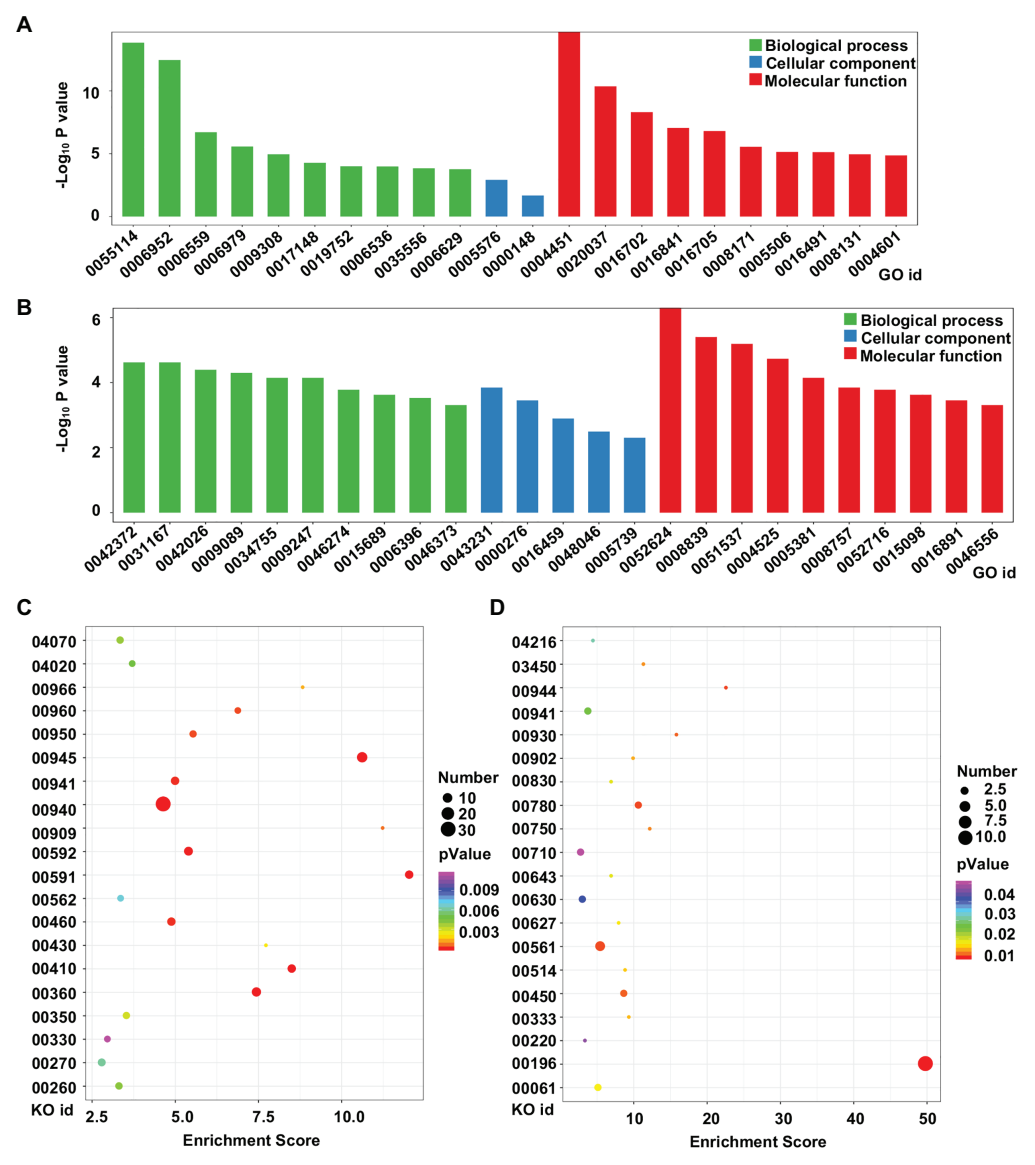
Functional analysis of DEGs between diploid and tetraploid barley. **(A)** Gene Ontology (GO) analysis of upregulated genes; **(B)** GO analysis of downregulated genes; **(C)** Kyoto Encyclopedia of Genes and Genomes (KEGG) analysis of upregulated genes; **(D)** KEGG analysis of downregulated genes. For the GO ids, 0055114: oxidation-reduction process; 0006952: defense response; 0006559: L-phenylalanine catabolic process; 0006979: response to oxidative stress; 0009308: amine metabolic process; 0017148: negative regulation of translation; 0019752: carboxylic acid metabolic process; 0006536: glutamate metabolic process; 0035556: intracellular signal transduction; 0006629: lipid metabolic process; 0005576: extracellular region; 0000148: 1,3-beta-D-glucan synthase complex; 0004451: isocitrate lyase activity; 0020037: heme binding; 0016702: oxidoreductase activity, acting on single donors with the incorporation of molecular oxygen, incorporation of two atoms of oxygen; 0016841: ammonia-lyase activity; 0016705: oxidoreductase activity, acting on paired donors, with incorporation or reduction of molecular oxygen; 0008171: O-methyltransferase activity; 0005506: iron ion binding; 0016491: oxidoreductase activity; 0008131: primary amine oxidase activity; 0004601: peroxidase activity; 0042372: phylloquinone biosynthetic process; 0031167: rRNA methylation; 0042026: protein refolding; 0009089: lysine biosynthetic process *via* diaminopimelate; 0034755: iron ion transmembrane transport; 0009247: glycolipid biosynthetic process; 0046274: lignin catabolic process; 0015689: molybdate ion transport; 0006396: RNA processing; 0046373: L-arabinose metabolic process; 0043231: intracellular membrane-bounded organelle; 0000276: mitochondrial proton-transporting ATP synthase complex, coupling factor F(o); 0016459: myosin complex; 0048046: apoplast; 0005739: mitochondrion; 0052624: 2-phytyl-1,4-naphthoquinone methyltransferase activity; 0008839: 4-hydroxy-tetrahydrodipicolinate reductase; 0051537: 2 iron, 2 sulfur cluster binding; 0004525: ribonuclease III activity; 0005381: iron ion transmembrane transporter activity; 0008757: S-adenosylmethionine-dependent methyltransferase activity; 0052716: hydroquinone: oxygen oxidoreductase activity; 0015098: molybdate ion transmembrane transporter activity; 0016891: endoribonuclease activity, producing 5'-phosphomonoesters; 0046556: alpha-L-arabinofuranosidase activity. For the KO ids, 04070: phosphatidylinositol signaling system; 04020: calcium signaling pathway; 00966: glucosinolate biosynthesis; 00960: tropane, piperidine and pyridine alkaloid biosynthesis; 00950: isoquinoline alkaloid biosynthesis; 00945: stilbenoid, diarylheptanoid and gingerol biosynthesis; 00941: flavonoid biosynthesis; 00940: phenylpropanoid biosynthesis; 00909: sesquiterpenoid and triterpenoid biosynthesis; 00592: alpha-linolenic acid metabolism; 00591: linoleic acid metabolism; 00562: inositol phosphate metabolism; 00460: cyanoamino acid metabolism; 00430: taurine and hypotaurine metabolism; 00410: beta-alanine metabolism; 00360: phenylalanine metabolism; 00350: tyrosine metabolism; 00330: arginine and proline metabolism; 00270: cysteine and methionine metabolism; 00260: glycine, serine, and threonine metabolism; 04216: ferroptosis; 03450: non-homologous end-joining; 00944: flavone and flavonol biosynthesis; 00930: caprolactam degradation; 00902: monoterpenoid biosynthesis; 00830: retinol metabolism; 00780: biotin metabolism; 00750: vitamin B6 metabolism; 00710: carbon fixation in photosynthetic organisms; 00643: styrene degradation; 00630: glyoxylate and dicarboxylate metabolism; 00627: aminobenzoate degradation; 00561: glycerolipid metabolism; 00514: other types of O-glycan biosynthesis; 00450: selenocompound metabolism; 00333: prodigiosin biosynthesis; 00220: arginine biosynthesis; 00196: photosynthesis-antenna proteins; 00061: fatty acid biosynthesis.

To analyze the potential functions of the DEGs in tetraploid barley relative to diploid barley, GO and KEGG analyses were conducted. GO analysis of upregulated genes determined that 22 terms were enriched across all the three categories, though mostly in those of biological process and molecular function, in which the three terms of oxidation-reduction process (GO id: 05114), defense response (GO id: 0006952), and isocitrate lyase activity (GO id: 0004451) were evidently the three most enriched (Figure 5A). There were 25 terms enriched for downregulated genes with similar characteristics of upregulated genes, while the three most enriched terms were 2-phytyl-1,4-naphthoquinone methyltransferase activity (GO id: 0052624), 4-hydroxy-tetrahydrodipicolinate reductase (GO id: 0008839), and the 2 iron, 2 sulfur cluster binding (GO id: 0051537; Figure 5B).

KEGG analysis indicated that the top-20 enriched pathways of upregulated genes were mainly related to metabolism or biosynthesis, with the three pathways of phenylpropanoid biosynthesis (KO id: 00940), stilbenoid, diarylheptanoid, and gingerol biosynthesis (KO id: 00945), and phenylalanine metabolism (KO id: 00360) harboring the most the most DEGs, while the linoleic acid metabolism (KO id: 00591), sesquiterpenoid, and triterpenoid biosynthesis (KO id: 00909), and stilbenoid, diarylheptanoid, and gingerol biosynthesis (KO id: 00945) pathways featured the highest rich factors (Figure 5C). The top-20 enriched pathways of downregulated genes were more varied and complicated; however, the pathway of photosynthesis-antenna proteins (KO id: 00196) included the most DEGs and had the highest rich factor (Figure 5D).

### Validation by qRT-PCR for RNA-Seq

**Figure 6:**
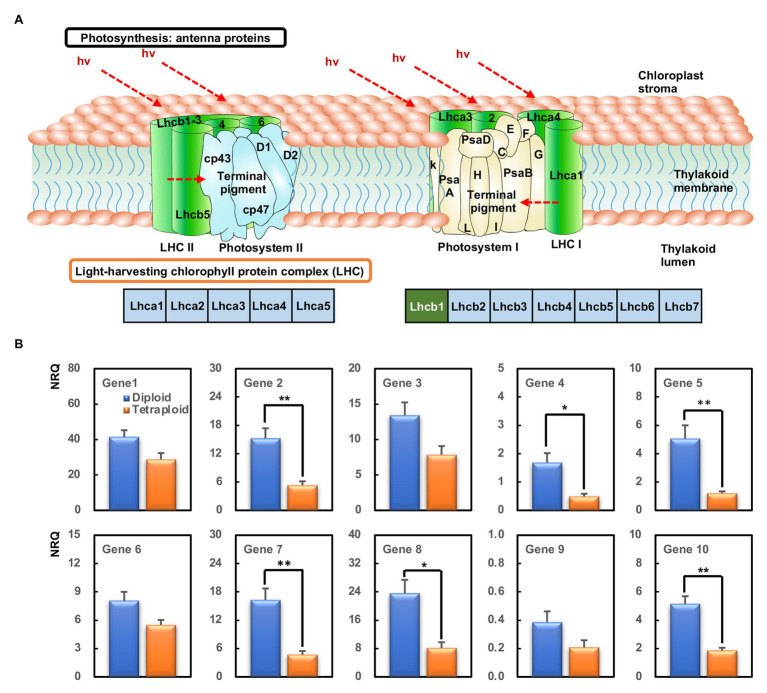
The most enriched KEGG pathways and genes’ expression analyzed by quantitative PCR (qPCR). **(A)** The KEGG pathway of photosynthesis-antenna proteins. The green box within Lhcb1 means those downregulated. **(B)** Relative gene expression [normalized relative quantity (NRQ)] of DEGs between the diploid and tetraploid barley. The ^**^ indicates significantly differential expression between the diploid and tetraploid barley in a *t*-test at 0.01 level and the ^*^ at 0.05 level (*n* = 3); Gene 1, *HORVU1Hr1G088900*; Gene 2, *HORVU1Hr1G088920*; Gene 3, *HORVU1Hr1G089180*; Gene 4, *HORVU6Hr1G016850*; Gene 5, *HORVU6Hr1G016880*; Gene 6, *HORVU6Hr1G016940*; Gene 7, *HORVU6Hr1G091650*; Gene 8, *HORVU6Hr1G091660*; Gene 9, *HORVU7Hr1G040370*; Gene 10, *HORVU7Hr1G040380*.

All the DEGs involved in the photosynthesis-antenna proteins, with the exception of *HORVU1Hr1G088930* because of its unstable detection by qPCR, were selected for validation by qPCR. These comprised *HORVU1Hr1G088900*, *HORVU1Hr1G088920*, *HORVU1Hr1G089180*, *HORVU6Hr1G016850*, *HORVU6Hr1G016880*, *HORVU6Hr1G016940*, *HORVU6Hr1G091650*, *HORVU6Hr1G091660*, *HORVU7Hr1G040370*, and *HORVU7Hr1G040380*, all of which were related to the Lhcb1 component (Figure 6A). These qPCR results confirmed all 10 genes were downregulated in the tetraploid barley compared with the diploid barley, albeit not always significantly, a pattern basically consistent with the corresponding RNA-Seq data (Figure 6B).

## Discussion

### Superior Characteristics of Tetraploid Barley for Organ Size and Photosynthesis

Polyploids often feature enlarged organs, including both vegetative and reproductive organs (Otto, 2007). In this research, we found that the leaves of tetraploid barley were thicker than those of diploid barley at this stage (which was confirmed by shoot dry weight), and tetraploid barley also had larger-sized stomata and seeds (Figures 1B,C; Supplementary Figure S2A; Supplementary Table S3). Yet, at a given growth time-point, tetraploid barley had fewer leaves than diploid barley, a finding also observed in *Stevia rebaudiana* plants (Xiang et al., 2019), and it was thought to arise from occasionally prolonged vegetative growth (Dudits et al., 2016). In addition, other cells were also enlarged alongside the bigger stomatal cells in leaves of tetraploid barley, which had lower stomatal density than those of diploid barley (Figure 2B). A similar trade-off was reported in work done by Padoan et al. (2013).

Chlorophyll is the key photosynthetic pigment in plants, playing critical roles in the photosynthesis of flowering plants (Masuda and Fujita, 2008; Cao et al., 2018). Here, we found that leaf chlorophyll contents of tetraploid barley were significantly increased over those of diploid barley, suggesting that the tetraploid barley might be superior at carrying out photosynthesis (Table 1). Further, diurnal variation in photosynthetic characteristics indicated tetraploid barley performed better than diploid barley in terms of P_n_, G_s_, and T_r_ and more C_i_ consumed, an interpretation confirmed by the light- and CO_2_-response P_n_ curves (Figures 2, 3). These results disagree with an early study of barley (Sicher et al., 1984), perhaps because controlled light conditions were used in that research. In our study of barley, the net photosynthetic rate in tetraploids showed significant improvement over diploids when exposed to intense light, but it was similar under exposure to low light. Additionally, the carotenoid contents were also augmented in tetraploid barley, indicating that it might also be superior at quenching the excessive excitation energy, which plays a crucial role in photoprotection combined with downregulation of Lhcb1-encoding genes (Table 1, Figure 6A).

Moreover, several photosynthetic parameters were significantly higher in tetraploid barley, namely P_max_, LSP, V_c,max_, and J_max_ (Table 2). The higher P_max_ and LSP suggested that tetraploid barley was better able to adjust to high light intensity, and the higher V_c,max_ and J_max_ implied tetraploid barley was also more active in carbon assimilation, especially the higher of J_max_ would be helpful for improving V_c,max_ (von Caemmerer and Farquhar, 1981). These results demonstrate that the photosynthetic capacity is stronger in tetraploid than diploid barley plants. It was also observed by Cao et al. (2018), although the V_c,max_ and J_max_ were not significantly higher in the tetraploid.

### Subtle Changes in Gene Expression Between Diploid and Tetraploid Barley

To investigate the differences of potential molecular mechanisms between the tetraploid and diploid barley, a transcriptome comparison was conducted for their leaves when photosynthesis was highest. We found that the genes’ expression of these six samples – three for the diploid barley and three for the tetraploid barley – very similar according to Spearman correlations and the PCA (Supplementary Figure S1), with only 793 DEGs uncovered (about 2.9% of all detected genes), indicating minor differences in gene expression between diploid and tetraploid barley overall. This agrees with the findings of several other reports (Lu et al., 2006; Pignatta et al., 2010; Gao et al., 2016; Xiang et al., 2019). Nevertheless, we did uncover a high proportion of upregulated DEGs (73.1%) that appeared among the identified DEGs *via* global transcriptomic profiling, a percentage higher than in those reports (Gao et al., 2016; Xiang et al., 2019). Further studies await to discern whether more potential genes were ploidy-responsive or activated in tetraploid barley.

### Downregulation of Antenna Protein-Encoding Genes in Tetraploid Barley

Light-harvesting antenna complexes and two reaction centers of photosystem II (PSII) and photosystem I (PSI) constitute the system of light-energy capture and photochemistry in natural photosynthesis (Liu and Blankenship, 2019). Antenna proteins are crucial for photosynthesis, by absorbing light, and their higher activity usually augments photosynthesis, especially when light is a limiting resource (Blankenship and Chen, 2013). But these proteins could be harmful at much higher light intensities, especially when the capacity of processing the intermediate products generated by the primary photochemistry lags too far behind (Blankenship and Chen, 2013), in what is known broadly as photoinhibition (Vass et al., 2007). We found that the KEGG pathway of photosynthesis-antenna proteins was significantly enriched, and all genes in this pathway were downregulated in barley. Considering the sampling time used, the higher light density was better for the photosynthesis while the higher temperature was harmful, so there should have been enough or even excess light for the photosynthesis during this time period. Accordingly, reducing the transcription of antenna protein-encoding genes around noon might be more beneficial for photosynthesis in the tetraploid barley compared with the diploid barley. Furthermore, these downregulated genes were all related to the Lhcb1 component in the PSII, indicating that the primary site of photoinhibition might lie within the PSII complex (Vass et al., 2007). We suggest that tetraploid barley harbors different regulation patterns to strike a balance between protection from high-light irradation and maintaining its high photosynthetic capacity.

## Conclusion

In this work, a homogenous double haploid and autotetraploid were rapidly produced in barley through *in vitro* microspore culturing with spontaneously chromosomal doubling. The tetraploid barley plants possess the distinct characteristic of polyploids in having a larger cellular or organ size (i.e., leaf). Photosynthetic capacity was enhanced in tetraploids *via* increased photosynthetic pigments and photosynthesis (especially under high light intensity). Furthermore, a transcriptomic analysis revealed that subtle changes of global gene expression occur in these tetraploids through small subset (793, ~2.3% of all detected genes) of identified DEGs, of which most (580; 73.1%) were upregulated. The KEGG enriched pathway was closed with the photosynthesis-antenna proteins, in which light-harvesting cholorophyII *a*/*b*-binding protein (Lhcb1) components were downregulated in the tetraploid samples. Taken together, our results provide evidences for understanding of enhanced photosynthetic capacity caused by polyploidization in morphology, photosynthetic physiology, and transcriptome in barley.

## Data Availability Statement

The original contributions presented in the study are publicly available. This data can be found at: NCBI with submission ID: SUB7735025 and bioProject ID: PRJNA642324.

## Author Contributions

YC performed RNA-Seq related experiments, including barley plants’ culturing, sample preparations, cDNA synthesis, and the qPCR. HX performed photochemistry- and photosynthesis-related experiments, including the analysis of photosynthesis pigments, measuring of photosynthetic parameters, and evaluating the light- and CO_2_-response curves. TH measured the stomatal guard cells’ length. RG conducted the cytological identification of barley chromosomes. GG and RL obtained and provided the stable diploid and tetraploid barley seeds from the microspore cultures. YC and HX prepared and wrote the manuscript. ZC supervised the experiments and revised the manuscript. CL designed the study. All authors contributed to the article and approved the submitted version.

### Conflict of Interest

The authors declare that the research was conducted in the absence of any commercial or financial relationships that could be construed as a potential conflict of interest.

## Figures and Tables

**Table:** 

Term	Definitions
AQY	Apparent quantum yield
C_a_	CO_2_ concentration
Car	Carotenoid
Chl *a*	Chlorophyll *a*
Chl *b*	Chlorophyll *b*
Chl (*a+b*)	Total chlorophyll
C_i_	Intercellular CO_2_ concentration
DEGs	Differentially expressed genes
FC	Fold change
FPKM	Fragments per kilo bases per million reads
F_v_’/F_m_’	The maximal photochemical efficiency of PSII in light
GO	Gene Ontology
G_s_	Stomatal conductance
HC	High confidence
J_max_	Maximum rate of electron transport
KEGG	Kyoto Encyclopedia of Genes and Genomes
LCP	Light compensation point
Lhcb1	Light-harvesting cholorophyII *a*/*b*-binding protein
LSP	Light saturation point
PCA	Principal component analysis
P_max_	Maximum net photosynthetic rate
P_n_	Net photosynthetic rate
PPFD	Photosynthetic photon flux density
PSI	Photosystem I
PSII	Photosystem II
T_r_	Transpiration rate
V_c,max_	Maximum RuBP saturated rate of carboxylation

## Footnotes

^1^http://plants.ensembl.org/Hordeum_vulgare/Info/Index?db=core

^2^http://webblast.ipk-gatersleben.de/barley_ibsc/downloads/Hv_IBSC_PGSB_r1_HighConf.gtf.gz

^3^http://geneontology.org/

^4^http://www.genome.jp/kegg/

## References

[ref1] Anders S. Pyl P. T. Huber W. (2015). HTSeq-a Python framework to work with high-throughput sequencing data. Bioinformatics 31, 166–169. 10.1093/bioinformatics/btu638, PMID: 25260700 PMC4287950

[ref2] Arias S. Bhatia S. K. (2015). “Barley” in Medical applications for biomaterials in Bolivia. Cham: Springer.

[ref3] Arnon D. (1949). Copper enzymes in isolated chloroplasts. Polyphenoloxidase in *Beta vulgaris*. Plant Physiol. 24, 1–15. 10.1104/pp.24.1.1, PMID: 16654194 PMC437905

[ref4] Blankenship R. E. Chen M. (2013). Spectral expansion and antenna reduction can enhance photosynthesis for energy production. Curr. Opin. Chem. Biol. 17, 457–461. 10.1016/j.cbpa.2013.03.031, PMID: 23602382

[ref600] Bolger A. M. Lohse M. Usadel B. (2014). Trimmomatic: a flexible trimmer for Illumina sequence data. Bioinformatics 30, 2114–2120. 10.1093/bioinformatics/btu170 24695404 PMC4103590

[ref5] Cao Q. Zhang X. Gao X. Wang L. Jia G. (2018). Effects of ploidy level on the cellular, photochemical and photosynthetic characteristics in *Lilium* FO hybrids. Plant Physiol. Biochem. 133, 50–56. 10.1016/j.plaphy.2018.10.027, PMID: 30390431

[ref6] Castillo A. M. Cistué L. Vallés M. P. Soriano M. (2009). Chromosome doubling in monocots. Advances in haploid production in higher plants. Netherlands: Springer.

[ref7] Chen Z. Huang J. Muttucumaru N. Powers S. J. Halford N. G. (2013). Expression analysis of abscisic acid (ABA) and metabolic signalling factors in developing endosperm and embryo of barley. J. Cereal Sci. 58, 255–262. 10.1016/j.jcs.2013.06.009, PMID: 24748715 PMC3990443

[ref8] Chen Z. Lu R. Zou L. Du Z. Gao R. He T.. (2012). Genetic diversity analysis of barley landraces and cultivars in the Shanghai region of China. Genet. Mol. Res. 11, 644–650. 10.4238/2012.March.16.2, PMID: 22535400

[ref9] Dong B. Wang H. Liu T. Cheng P. Chen Y. Chen S.. (2017). Whole genome duplication enhances the photosynthetic capacity of Chrysanthemum nankingense. Mol. Gen. Genomics. 292, 1247–1256. 10.1007/s00438-017-1344-y, PMID: 28674743

[ref10] Dudits D. Török K. Cseri A. Paul K. Nagy A. V. Nagy B.. (2016). Response of organ structure and physiology to autotetraploidization in early development of energy willow *Salix viminalis*. Plant Physiol. 170, 1504–1523. 10.1104/pp.15.01679, PMID: 26729798 PMC4775130

[ref11] Eliška J. Vladimíra N. H. Martin D. (2015). Photosynthetic characteristics of three ploidy levels of *Allium oleraceum* L. (Amaryllidaceae) differing in ecological amplitude. Plant Spec. Biol. 30, 212–224. 10.1111/1442-1984.12053

[ref12] Farquhar G. D. von Caemmerer S. Berry J. A. (1980). A biochemical model of photosynthetic CO_2_ assimilation in leaves of C3 species. Planta 149, 78–90. 10.1007/BF00386231, PMID: 24306196

[ref13] Gao R. Guo G. Fang C. Huang S. Chen J. Lu R.. (2018). Rapid generation of barley mutant lines with high nitrogen uptake efficiency by microspore mutagenesis and field screening. Front. Plant Sci. 9:450. 10.3389/fpls.2018.00450, PMID: 29681915 PMC5897737

[ref14] Gao R. Wang H. Dong B. Yang X. Chen S. Jiang J.. (2016). Morphological, genome and gene expression changes in newly induced autopolyploid *Chrysanthemum lavandulifolium* (Fisch. ex Trautv.) Makino. Int. J. Mol. Sci. 17:1690. 10.3390/ijms17101690, PMID: 27735845 PMC5085722

[ref15] Gao S. Yan Q. Chen L. Song Y. Li J. Fu C.. (2017). Effects of ploidy level and haplotype on variation of photosynthetic traits: novel evidence from two *Fragaria* species. PLoS One 12:e0179899. 10.1371/journal.pone.0179899, PMID: 28644876 PMC5482484

[ref16] He T. Guo G. Chen Z. Du Z. Gao R. Xu H.. (2014). Relationship between the stomatal guard cell length and ploidy level in barley microspore regenerated plantlets. J. Triticeae Crops 34, 175–180. 10.7606/j.issn.1009-1041.2014.02.06

[ref17] Hejnák V. Hniličková H. Hnilička F. Andr J. (2016). Gas exchange and *Triticum* sp. with different ploidy in relation to irradiance. Plant Soil Environ. 62, 47–52. 10.17221/591/2015-PSE

[ref18] Hu F. Liu H. Wang F. Bao R. Liu G. (2015). Root tip chromosome karyotype analysis of hyacinth cultivars. Genet. Mol. Res. 14, 10863–10876. 10.4238/2015.September.9.24, PMID: 26400314

[ref19] Kim D. Langmead B. Salzberg S. L. (2015). HISAT: a fast spliced aligner with low memory requirements. Nat. Methods 12, 357–360. 10.1038/nmeth.3317, PMID: 25751142 PMC4655817

[ref20] Laere K. V. França S. C. Vansteenkiste H. Huylenbroeck J. V. Steppe K. Labeke M. C. V. (2011). Influence of ploidy level on morphology, growth and drought susceptibility in *Spathiphyllum wallisii*. Acta Physiol. Plant. 33, 1149–1156. 10.1007/s11738-010-0643-2

[ref21] Li Y. Guo G. Zhou L. Chen Y. Zong Y. Huang J.. (2019). Transcriptome analysis identifies candidate genes and functional pathways controlling the response of two contrasting barley varieties to powdery mildew infection. Int. J. Mol. Sci. 21:151. 10.3390/ijms21010151, PMID: 31878350 PMC6982059

[ref22] Liu H. Blankenship R. E. (2019). On the interface of light-harvesting antenna complexes and reaction centers in oxygenic photosynthesis. Bioenergetics 1860:148079. 10.1016/j.bbabio.2019.148079, PMID: 31518567

[ref23] Liu B. Li M. Li Q. Cui Q. Zhang W. Ai X.. (2018). Combined effects of elevated CO_2_ concentration and drought stress on photosynthetic performance and leaf structure of cucumber (*Cucumis sativus* L.) seedlings. Photosynthetica 56, 942–952. 10.1007/s11099-017-0753-9

[ref24] Long S. P. Bernacchi C. J. (2003). Gas exchange measurements, what can they tell us about the underlying limitations to photosynthesis? Procedures and sources of error. J. Exp. Bot. 54, 2393–2401. 10.1093/jxb/erg262, PMID: 14512377

[ref25] Lu B. Pan X. Zhang L. Huang B. Sun L. Li B.. (2006). A genome-wide comparison of genes responsive to autopolyploidy in *Isatis indigotica* using *Arabidopsis thaliana* affymetrix genechips. Plant Mol. Biol. Report. 24, 197–204. 10.1007/BF02914058

[ref26] Lu R. Wang Y. Sun Y. Shan L. Chen P. Huang J. (2008). Improvement of isolated microspore culture of barley (*Hordeum vulgare* L.): the effect of floret co-culture. Plant Cell Tissue Organ Cult. 93, 21–27. 10.1007/s11240-008-9338-4

[ref27] Masuda T. Fujita Y. (2008). Regulation and evolution of chlorophyll metabolism. Photochem. Photobiol. Sci. 7, 1131–1149. 10.1039/b807210h, PMID: 18846277

[ref28] Newman C. W. Newman R. K. (2006). A brief history of barley foods. Cereal Foods World 51, 4–7. 10.1094/CFW-51-0004

[ref29] Otto S. P. (2007). The evolutionary consequences of polyploidy. Cell 131, 452–462. 10.1016/j.cell.2007.10.022, PMID: 17981114

[ref30] Padoan D. Mossad A. Benedetta C. Chiancone B. Germana M. A. Khan P. S. S. V. (2013). Ploidy levels in citrus clementine affects leaf morphology, stomatal density and water content. Theor. Exp. Plant Phys. 25, 283–290. 10.1590/S2197-00252013000400006

[ref31] Pignatta D. Dilkes B. P. Yoo S. Y. Henry I. M. Madlung A. Doerge R. W.. (2010). Differential sensitivity of the *Arabidopsis thaliana* transcriptome and enhancers to the effects of genome doubling. New Phytol. 186, 194–206. 10.1111/j.1469-8137.2010.03198.x, PMID: 20409178

[ref32] Quan X. Zeng J. Ye L. Chen G. Han Z. Shah J.. (2016). Transcriptome profiling analysis for two Tibetan wild barley genotypes in responses to low nitrogen. BMC Plant Biol. 16:30. 10.1186/s12870-016-0721-8, PMID: 26817455 PMC4728812

[ref33] Ramakers C. Ruijter J. M. Deprez R. H. L. Moorman A. F. M. (2003). Assumption-free analysis of quantitative real-time polymerase chain reaction (PCR) data. Neurosci. Lett. 339, 62–66. 10.1016/S0304-3940(02)01423-4, PMID: 12618301

[ref34] Roberts A. Pimentel H. Trapnell C. Pachter L. (2011). Identification of novel transcripts in annotated genomes using RNA-Seq. Bioinformatics 27, 2325–2329. 10.1093/bioinformatics/btr355, PMID: 21697122

[ref35] Sattler M. C. Carvalho C. R. Clarindo W. R. (2016). The polyploidy and its key role in plant breeding. Planta 243, 281–296. 10.1007/s00425-015-2450-x, PMID: 26715561

[ref36] Sicher R. C. Kremer D. F. Harris W. G. Baenziger P. S. (1984). Photosynthate partitioning in diploid and autotetraploid barley (*Hordeum vulgare*). Physiol. Plant. 60, 239–246. 10.1111/j.1399-3054.1984.tb04571.x

[ref37] Soriano M. Cistué L. Vallés M. P. Castillo A. M. (2007). Effects of colchicine on anther and microspore culture of bread wheat (*Triticum aestivum* L.). Plant Cell Tiss. Org. 91, 225–234. 10.1007/s11240-007-9288-2

[ref38] Sun Q. Sun H. Bell R. Li L. Zhou G. Xin L.. (2015). Field performance of vegetative form traits of neopolyploids produced by in vitro colchicine treatment in *Pyrus communis*. Sci. Hortic-Amsterdam. 193, 182–187. 10.1016/j.scienta.2015.06.047

[ref500] Porra R. J. (2002). The chequered history of the development and use of simultaneous equations for the accurate determination of chlorophylls a and b. Photosynth. Res. 73, 149–156. 10.1023/A:1020470224740, PMID: 16245116

[ref39] Vass I. Cser K. Cheregi O. (2007). Molecular mechanisms of light stress of photosynthesis. Ann. N. Y. Acad. Sci. 1113, 114–122. 10.1196/annals.1391.017, PMID: 17513459

[ref40] von Caemmerer S. Farquhar G. D. (1981). Some relationships the biochemistry of photosynthesis and the gas exchange of leaves. Planta 153, 376–387. 10.1007/BF00384257, PMID: 24276943

[ref41] Vyas P. Bisht M. S. Miyazawa S. -I. Yano S. Noguchi K. Terashima I.. (2007). Effects of polyploidy on photosynthetic properties and anatomy in leaves of *Phlox drummondii*. Funct. Plant Biol. 34, 673–682. 10.1071/FP07020, PMID: 32689395

[ref42] Xiang Z. Tang X. Liu W. Song C. (2019). A comparative morphological and transcriptomic study on autotetraploid *Stevia rebaudiana* (Bertoni) and its diploid. Plant Physiol. Biochem. 143, 154–164. 10.1016/j.plaphy.2019.09.003 31505448

[ref43] Xu H. Liu C. Lu R. Guo G. Chen Z. He T.. (2016). The difference in responses to nitrogen deprivation and re-supply at seedling stage between two barley genotypes differing nitrogen use efficiency. Plant Growth Regul. 79, 119–126. 10.1007/s10725-015-0117-z

[ref44] Xue H. Zhang B. Tian J. Chen M. Zhang Y. Zhang Z.. (2017). Comparison of the morphology, growth and development of diploid and autotetraploid ‘Hanfu’ apple trees. Sci. Hortic-Amsterdam. 225, 277–285. 10.1016/j.scienta.2017.06.059

[ref45] Yuan S. Su Y. Liu Y. Li Z. Fang Z. Yang L.. (2015). Chromosome doubling of microspore-derived plants from cabbage (*Brassica oleracea var. capitata* L.) and broccoli (*Brassica oleracea var. italica* L.). Front. Plant Sci. 6:1118. 10.3389/fpls.2015.01118, PMID: 26734028 PMC4686604

[ref46] Zhang X. Cao Q. Jia G. (2017). A protocol for fertility restoration of F_1_ hybrid derived from *Lilium*×*formolongi* ‘Raizan 3’×oriental hybrid ‘Sorbonne’. Plant Cell Tiss. Org. 129, 375–386. 10.1007/s11240-017-1184-9

