# Supplementary material for: Comparative Analysis of Morphology, Photosynthetic Physiology, and Transcriptome Between Diploid and Tetraploid Barley Derived From Microspore Culture
Source: Front Plant Sci. 2021 Feb 26;12:626916. doi: 10.3389/fpls.2021.626916 (PMC7970760; doi:10.3389/fpls.2021.626916)
Supplement: Supplementary file 1 [file Data_Sheet_1.docx]

**Table S1.** Summary of RNA-seq data from barley shoots of the diploid and tetrploid barley (n=3).

|  | Diploid | | |  | Tetrploid | | |
| --- | --- | --- | --- | --- | --- | --- | --- |
|  | D-1 | D-2 | D-3 |  | T-1 | T-2 | T-3 |
| Raw bases (Gb) | 7.41 | 7.41 | 7.41 |  | 7.41 | 7.41 | 7.41 |
| Clean bases (Gb) | 6.73 | 6.82 | 6.77 |  | 6.74 | 6.75 | 6.87 |
| Q30 (%) | 92.75 | 93.28 | 93.15 |  | 92.93 | 92.79 | 93.24 |
| GC (%) | 56.09 | 56.72 | 56.39 |  | 56.83 | 56.63 | 56.54 |

**Table S2.** Details of the eleven differential expressed genes and nucleotide sequences of primers used in the qRT-PCR analyses.

| Gene Id/name | Description/annotation | Primer nucleotide sequence (5′-3′) | Amplicon (bp) | PCR efficiency | Origin |
| --- | --- | --- | --- | --- | --- |
| HORVU1Hr1G088900 | Chlorophyll a-b binding protein, chloroplastic | F: CGGCTGATCCTGAGACCTTC | 193 | 1.774 | This study |
|  |  | R: GGGTTGCCAAGGTAGTCGAG |  |  |  |
| HORVU1Hr1G088920 |  | F: AAGGCGGTGAAGAATGTCCC | 131 | 1.798 |  |
|  |  | R: AGGTAGAGCACACGATCAGC |  |  |  |
| HORVU1Hr1G089180 |  | F: CCCTGAAGAACTTGCCCTCG | 197 | 1.754 |  |
|  |  | R: GGTGTCCCATCCGTAGTCAC |  |  |  |
| HORVU6Hr1G016850 |  | F: ATCGTCAACTCTCTTCGGCG | 179 | 1.793 |  |
|  |  | R: TGTCCCACCCATAGTCACCA |  |  |  |
| HORVU6Hr1G016880 |  | F: GAGATCAAGAACGGTCGCCT | 148 | 1.777 |  |
|  |  | R: CAAAGTTGGTAGCGAACGCC |  |  |  |
| HORVU6Hr1G016940 |  | F: GACAACCTCGCTGACCACAT | 83 | 1.784 |  |
|  |  | R: CCTTACTTGCCGGGGACAAA |  |  |  |
| HORVU6Hr1G091650 |  | F: GCTGATCGTGTGCTCTACCT | 198 | 1.798 |  |
|  |  | R: CAACTCAGGGAACACGCA |  |  |  |
| HORVU6Hr1G091660 |  | F: GCTGATCGTGTGCTCTACCT | 198 | 1.777 |  |
|  |  | R: CAACTCAGGGAACACGCAAC |  |  |  |
| HORVU7Hr1G040370 |  | F: TGCTGATCGTGTGCTCTACC | 168 | 1.811 |  |
|  |  | R: AGCATAGCCCATCGACAGTG |  |  |  |
| HORVU7Hr1G040380 |  | F: CCTCGACTATCTCGGCAACC | 81 | 1.793 |  |
|  |  | R: CCCATGAGCACAACTTGGCA |  |  |  |
| GAPDH | glycolytic glyceraldehyde-3-phosphate dehydrogenase | F: AAGCATGAAGATACAGGGAGTGTG | 74 | 1.769 | Quan et al., 2016 |
|  |  | R: AATTTATTCTCGGAAGAGGTTGTACA |  |  |  |

**Table S3.** The length and width of seed and shoot dry weight of diploid and tetraploid barley

| Ploidy | Seed length (mm) | Seed width (mm) | Shoot dry weight (g) |
| --- | --- | --- | --- |
| Diploid | 9.05±0.65a | 3.05±0.26a | 0.1045±0.0199a |
| Tetraploid | 11.22±0.84b | 4.01±0.36b | 0.1380±0.0374b |

Values represent the mean ± standard deviation (n=30 for seed length and seed width, while n=15 for shoot dry weight). Different letters indicate significant differences between the diploid and tetraploid barley for each trait.

**
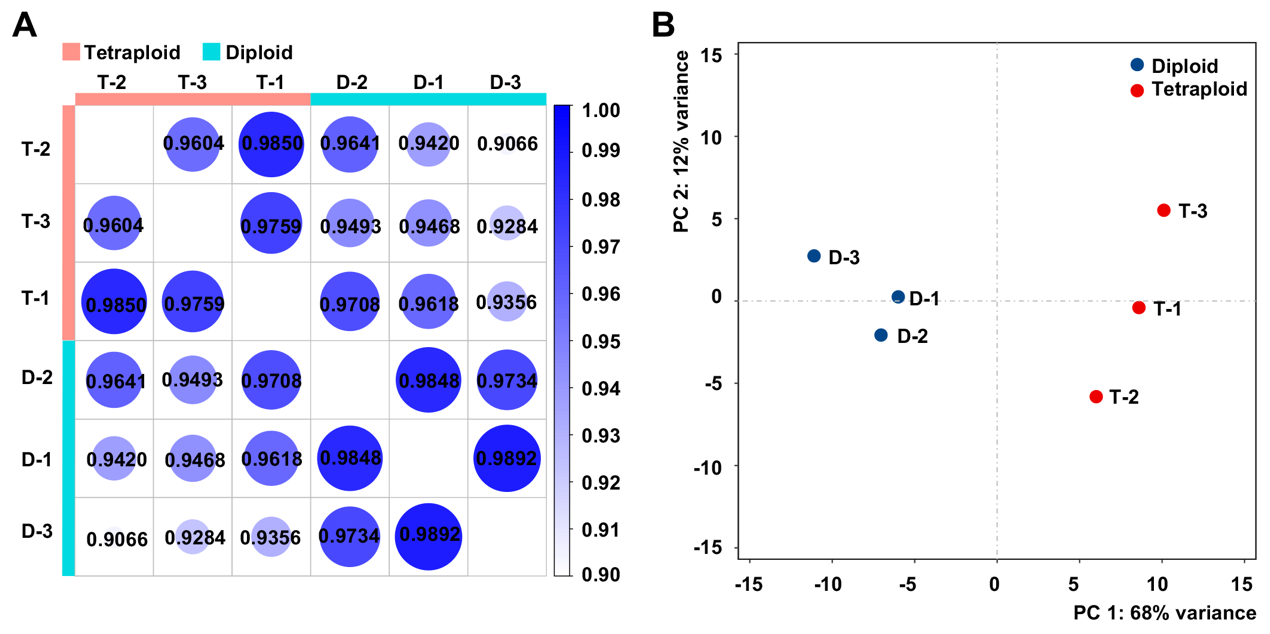
**

**Figure S1.** Spearman correlations and principal component analysis (PCA) for gene expressions of all samples. A. Spearman correlations. B. PCA. D-1,2,3 represent three biological replicates of the diploid barley; T-1,2,3 represent three biological replicates of the tetraploid barley.


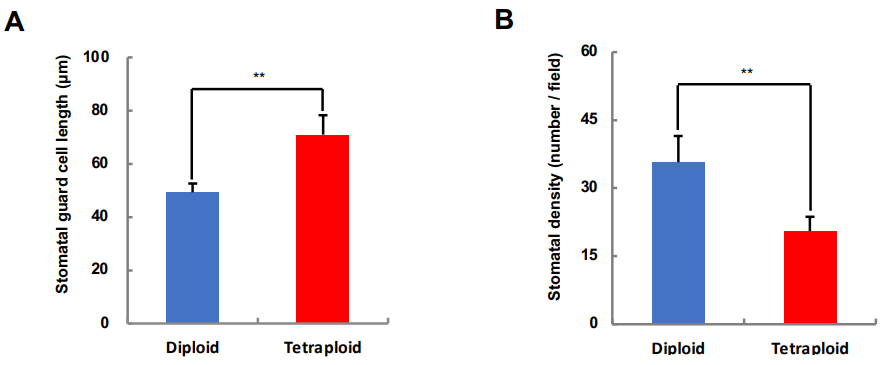


**Figure S2.** The length of stomatal guard cell and stomatal density of the diploid and teraploid barley. A. The length of stomatal guard cell. B. The stomatal density. “**” represents significant difference between the diploid and tetraploid barley at 0.01 level by t test (n=55 for the stomatal guard cell length, while n=15 for the stomatal density).
